# Boosting the Self-Trapped Exciton Emission in Cs_4_SnBr_6_ Zero-Dimensional Perovskite via Rapid Heat Treatment

**DOI:** 10.3390/nano13152259

**Published:** 2023-08-06

**Authors:** Haixia Wu, Zhenxu Lin, Jie Song, Yi Zhang, Yanqing Guo, Wenxing Zhang, Rui Huang

**Affiliations:** School of Materials Science and Engineering, Hanshan Normal University, Chaozhou 521041, Chinayqguo126@126.com (Y.G.);

**Keywords:** photoluminescence, self-trapped exciton, Cs_4_SnBr_6_, rapid thermal treatment

## Abstract

Zero-dimensional (0D) tin halide perovskites feature extraordinary properties, such as broadband emission, high photoluminescence quantum yield, and self-absorption-free characteristics. The innovation of synthesis approaches for high-quality 0D tin halide perovskites has facilitated the flourishing development of perovskite-based optoelectronic devices in recent years. However, discovering an effective strategy to further enhance their emission efficiency remains a considerable challenge. Herein, we report a unique strategy employing rapid heat treatment to attain efficient self-trapped exciton (STE) emission in Cs_4_SnBr_6_ zero-dimensional perovskite. Compared to the pristine Cs_4_SnBr_6_, rapid thermal treatment (RTT) at 200 °C for a duration of 120 s results in an augmented STE emission with the photoluminescence (PL) quantum yield rising from an initial 50.1% to a substantial 64.7%. Temperature-dependent PL spectra analysis, Raman spectra, and PL decay traces reveal that the PL improvement is attributed to the appropriate electron–phonon coupling as well as the increased binding energies of STEs induced by the RTT. Our findings open up a new avenue for efficient luminescent 0D tin-halide perovskites toward the development of efficient optoelectronic devices based on 0D perovskites.

## 1. Introduction

All-inorganic cesium lead halide perovskite quantum dots (QDs), denoted as CsPbX_3_ (X = Cl, Br, and I), are characterized by outstanding optical attributes, including high photoluminescence quantum yields (PL QYs) and narrow emission line widths. These properties render them as promising candidates for diverse applications encompassing photodetectors, low-threshold lasers, light-emitting diodes, and high-definition displays [1]. Nevertheless, their susceptibility to degradation under exposure to humid air, ultraviolet radiation, and elevated temperatures poses a significant challenge to their practical deployment [2]. In recent years, zero-dimensional (0D) metal halides have attracted significant attention due to their remarkable PL QYs and adjustable emissions. These materials, characterized by the confinement of luminescent metal halide octahedra by organic or inorganic cations, offer great potential for various optoelectronic applications, including LEDs, solar cells, scintillators, sensors, and thermal imaging systems [3,4,5]. In these materials, the Jahn–Teller distortion of metal halide octahedra upon photoexcitation leads to the localization of excitons, enhancing radiative emission by preventing exciton migration to defects. This phenomenon, known as the self-trapping of excitons (STEs), contributes to high PL QY and improved stability [6,7]. While significant progress has been made in exploring efficient 0D metal halides, the use of lead-based compounds such as Cs_4_PbBr_6_ is limited by the toxicity of lead, posing environmental and health concerns [8,9,10]. To address this issue, tin (Sn^2+^) has emerged as a promising alternative to lead (Pb^2+^) due to its similar electronic properties and environmentally friendly nature [11,12,13]. Numerous studies have probed into the intriguing properties of 0D tin halide perovskites, including Cs_4_SnX_6_ (where X = Br, I), and demonstrated their potential for optoelectronic applications [14,15,16]. For instance, the work conducted by Kovalenko and his team presented the compelling discovery of an efficient green-yellow emission derived from self-trapped excitons in Cs_4_SnBr_6_, with the achievement of a notable PL QY of 15 ± 5% at room temperature [14]. Intriguingly, through strategic substitution of Cs^+^ with Rb^+^ or K^+^ and Br^−^ with I^−^, both the PL peak position and Stokes shift can be simultaneously adjusted [14]. Zhang et al. introduced a novel room-temperature antisolvent method for synthesizing all-inorganic, lead-free green emissive Cs_4_SnBr_6_ 0D perovskites. These perovskites boast a PL QY of 20%, along with notable photostability and superior air stability in comparison to CsSnBr_3_ nanocrystals [15]. Quan et al. synthesized high-quality Cs_4_SnX_6_ (X = Br, I) nanocrystals that exhibit well-defined shapes and narrow size distributions, culminating in an impressive PL QY of up to 21% for Cs_4_SnBr_6_ nanocrystals [16]. However, a persistent obstacle facing tin halide perovskites is their lack of stability in air, a problem primarily attributed to the oxidation of Sn^2+^ to Sn^4+^, leading to a consequential decline in luminescent performance [17,18]. To overcome this issue, Zhang et al. implemented an innovative solution by introducing SnF_2_ as a tin source, which replaced the easily oxidizable SnBr_2_ and successfully enhanced the structural stability of Cs_4_SnBr_6_ perovskite by utilizing fluorine to suppress Sn^2+^ oxidation effectively [19]. Despite the remarkable PL performance displayed by 0D tin halide perovskites, the quest for their commercialization necessitates considerable efforts to further enhance both their PL efficiency and stability.

0D perovskites generally display highly efficient wide-band emission with a large Stokes shift. Numerous researchers have employed the STE model to elucidate the wide-band emission and large Stokes shift induced by octahedral distortions in 0D perovskites upon light excitation [20,21,22,23,24]. Strong electron–phonon coupling and a malleable lattice are widely considered the cornerstone factors driving the emission of STEs. Particularly within the context of 0D perovskites, excitons, when photoexcited, induce swift distortions in the lattice of the excited state, culminating in the formation of localized STEs [22,23,24,25,26]. Previous investigations have revealed that the lattice distortion in zero-dimensional metal halide perovskites can be manipulated by adjusting factors such as the chemical composition, temperature, and pressure. Such a modulation enables precise control of the STE states, which in turn optimizes luminescence performance [22,23,24,25,26,27]. For example, the introduction of varying metal ions into 0D metal halide perovskites can amplify the distortion of [BX_6_]^4−^ octahedra and bolster electron–phonon coupling, consequently enhancing the density of STE states and boosting luminescence efficiency [26]. Han et al. pioneered the study on all-inorganic, lead-free, 0D perovskite single crystals, specifically A_2_InCl_5_(H_2_O) (A = Rb, Cs), which featured a striking enhancement in yellow emission. They introduced Sb^3+^ doping into A_2_InCl_5_ (H_2_O), triggering a significant surge in PL QY from a modest 2% to an impressive 85–95%. The Sb-doped 0D rubidium indium chloride perovskites also exhibited remarkable stability. In a different vein, Ma’s team revealed an unexpected phenomenon in Cs_4_PbBr_6_ nanocrystals: a pressure-induced emission at room temperature when the pressure reaches 3.01 GPa. This emission under pressure is attributed to the amplified optical activity and increased binding energy of self-trapped excitons (STEs) in the high-pressure phase. The cause of this is thought to be the substantial distortion and increased rigidity of [PbBr_6_]^4−^ octahedra upon compression. In previous work, we successfully expanded the emission spectra and amplified the emission efficiency of STEs in Cs_4_SnBr_6_ through an innovative Mn^2+^ doping strategy [28]. This approach imbued the Mn^2+^-doped Cs_4_SnBr_6_ with remarkably enhanced PL QY of up to ~75.8%, a broader emission spectrum, and increased thermal stability. Combined experimental observations and first-principles calculations elucidate that the significant enhancement in PL is primarily a result of increased electron–phonon coupling and heightened binding energies of STEs, driven by the substantial distortion of [SnBr_6_]^4−^ octahedra triggered by Mn^2+^ doping. Furthermore, the color tuning of Mn^2+^-doped Cs_4_SnBr_6_ can be manipulated through the competitive transfers of free excitons to STE states and Mn^2+^ sites.

Rapid thermal treatment (RTT) is a widely used technique to modify the micro-nanostructure and enhance the optoelectronic properties of materials [29]. RTT involves rapid heating and cooling, characterized by short heating times and accelerated cooling rates. This transient process can induce changes in the structural order of metal halide octahedra, which plays an important role in the formation of STEs. However, the influence of RTT on the PL properties of 0D Cs_4_SnBr_6_ has not been fully explored. In this study, we propose a novel strategy based on RTT to enhance the emission of STEs in lead-free Cs_4_SnBr_6_. We systematically investigate the effects of RTT on the optical properties of Cs_4_SnBr_6_. Compared to pristine Cs_4_SnBr_6_, we find that RTT at 200 °C for 120 s leads to enhanced STE emission, with the PL QY escalating from an initial 50.1% to a substantial 64.7%. Temperature-dependent PL spectra analysis, Raman spectra, and PL decay traces reveal that the PL improvement is attributed to the appropriate electron–phonon coupling as well as the increased binding energies of STEs induced by the RTT.

## 2. Materials and Methods

To obtain stable Cs_4_SnBr_6_ perovskite, cesium bromide (CsBr, 99.9%, Aladdin, Shanghai, China), stannous fluoride (SnF_2_, 99.9%, Macklin, Shanghai, China), and ammonium bromide (NH_4_Br, 99.9%, Aladdin, Shanghai, China) were employed as reactant precursors to synthesize the SnF_2_-derived Cs_4_SnBr_6_ (Cs_4_SnBr_6_) using water-assisted wet ball-milling [19]. To achieve Cs_4_SnBr_6_, the molar ratios of CsBr, SnF_2_, and NH_4_Br were maintained at 4, 1, and 2 mmol, respectively. Initially, the precursors were loaded into a nylon ball-milling jar and combined with 60 μL of deionized water and zirconia balls. Subsequently, a ball milling process was conducted using a ball grinder (MSK-SFM-3-II, Hefei Kejing Material Technology Co., Ltd., Hefei, China) for 30 min at a speed of 600 rpm. The resulting product was then dried in a vacuum-drying oven for 120 min at room temperature and annealed at various temperatures ranging from 100 to 300 °C using a straightforward RTT process. The RTT process was executed on a rapid thermal processor, heating the sample to the annealing temperature at a rate of 10 °C s^−1^. After maintaining the annealing temperature for 30, 90, and 120 s, the system was rapidly cooled to room temperature. Upon cooling, the Cs_4_SnBr_6_ powder was acquired via ball milling for 30 min at a speed of 600 rpm. PL measurements were conducted using an Edinburgh Instrument FLS1000 PL spectrometer (Livingstone, Scotland). The PL spectra were obtained at different temperatures to investigate the temperature-dependent behavior. PL excitation (PLE) spectra and time-resolved PL spectra were also recorded using the same instrument. An excitation wavelength of 375 nm (375 nm, 70 ps excitation pulses LASER) was used to measure the time-resolved PL spectra. The crystal structures of Cs_4_SnBr_6_ were analyzed using X-ray diffraction (XRD) with a Bruker D8 Advance instrument (Karlsruhe, Germany). XRD measurements were performed at 35 kV and 35 mA to determine the crystal structure of the samples. The compositions of Cs_4_SnBr_6_ were determined through energy-dispersive spectroscopy (EDS) using a Bruker EDS QUANTAX system (Karlsruhe, Germany). Scanning electron microscopy (SEM) (a Hitachi SU5000 SEM instrument, Tokyo, Japan) was employed to investigate the surface morphology and microstructure of Cs_4_SnBr_6_.

## 3. Results and Discussion

**Figure 1:**
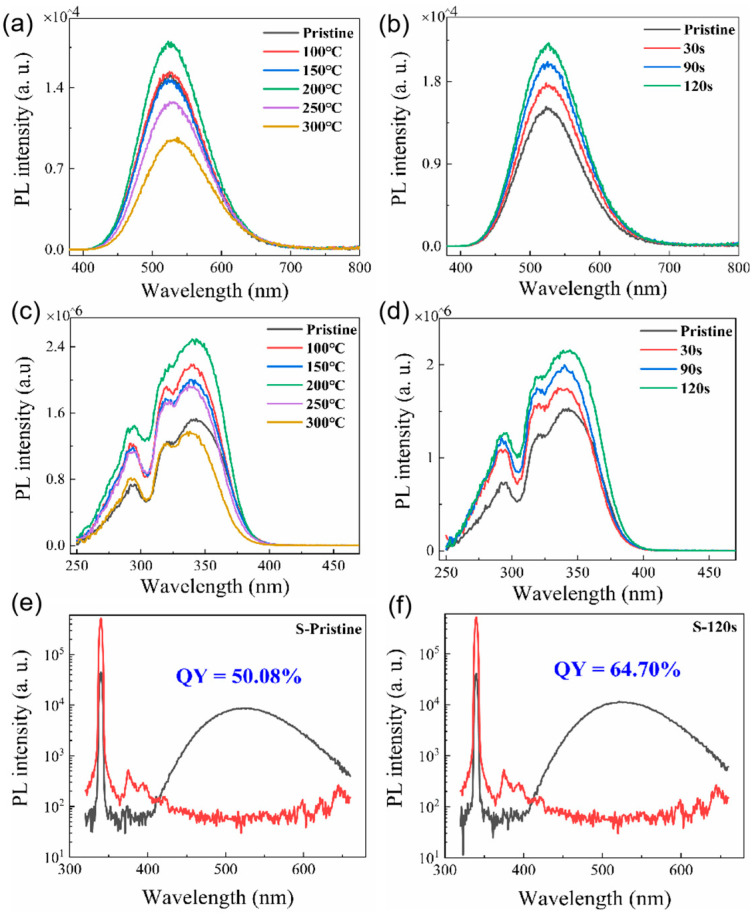
(**a**) PL spectra and (**c**) PLE spectra of both the unprocessed Cs_4_SnBr_6_ sample and samples treated with RTT at varying temperatures for a span of 30 s. (**b**) PL spectra and (**d**) PLE spectra of Cs_4_SnBr_6_ samples annealed at an RTT temperature of 200 °C for distinct durations. The PL spectra are excited by the 340 nm line from Xe lamp. The PL QY derived from the excitation and emission spectra for (**e**) the pristine Cs_4_SnBr_6_ and (**f**) Cs_4_SnBr_6_ sample annealed at an RTT temperature of 200 °C for a span of 120 s.

**Figure 2:**
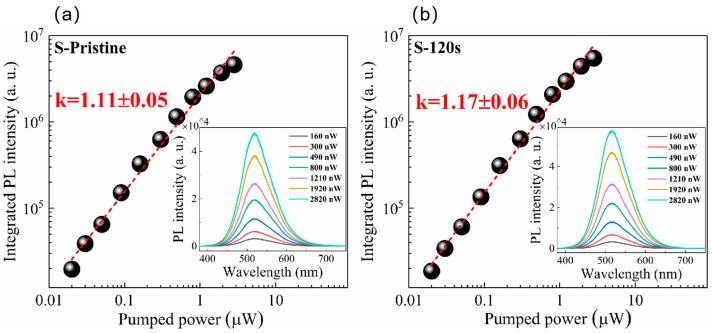
The integrated PL intensity under different excitation powers: (**a**) pristine sample, (**b**) sample S-120 s. The red solid lines are theoretical fitting curves. Insets in (**a**,**b**) show the PL spectra from pristine sample and sample S-120 s under different excitation powers, respectively.

The PL spectra of the pristine Cs_4_SnBr_6_ sample and samples subjected to RTT at different temperatures are shown in Figure 1a. Both types of samples exhibited a broad emission with a peak at approximately 530 nm. The emission band had a large full-width at half-maximum (FWHM) of approximately 105 nm (473 meV). Additionally, a significant Stokes shift of around 1.30 eV was observed, as illustrated in Figure 1c,d. It is evident that RTT at temperatures below 150 °C had minimal influence on the PL intensity of Cs_4_SnBr_6_ samples. However, a notable increase in PL intensity was observed when the RTT temperature was raised to 200 °C. Conversely, as the RTT temperature was further increased to 300 °C, the PL intensity of Cs_4_SnBr_6_ samples decreased rapidly. Figure 1b showcases PL spectra of Cs_4_SnBr_6_ samples annealed at an RTT temperature of 200 °C over diverse durations. The observed PL intensity of the Cs_4_SnBr_6_ samples appears to increment gradually with the prolongation of RTT duration from 30 to 120 s. Notably, as depicted in Figure 1e,f, the PL QY takes a significant leap from 50.1 to 64.7% following the annealing of the unprocessed Cs_4_SnBr_6_ at an RTT temperature of 200 °C for a span of 120 s. Figure 2a,b display the excitation power dependence of PL for both the pristine sample and sample S-120 s. The insets of Figure 2a,b demonstrate that an increase in excitation power, from 160 to 2820 nW, is accompanied by a corresponding enhancement in PL intensity. Nevertheless, the PL peak position remains consistent, unaffected by variations in the excitation power. Additionally, a distinct linear correlation emerges between the integrated PL intensity and the excitation power within the range of 160–2820 nW. The excitation power-dependent PL intensity is commonly employed to determine the underlying mechanism of light emission in semiconductors. As per the literature [29,30], the PL intensity (I) can be described by the equation $I={\eta I}_{0}^{k}$, where I_0_ denotes the excitation power, η symbolizes the emission efficiency, and the exponent k is affiliated with the radiative recombination process. A linear fit of ln(I/η) in contrast to ln(I_0_) allows the estimation of the k parameter values as 1.11 and 1.17 for the pristine sample and sample S-120 s, respectively. This deduction strongly suggests that the green emission in both samples is engendered by the recombination of excitons. Given the large Stokes shift of ~1.30 eV, coupled with a broad FWHM of the emission band approximating ~105 nm, and further considering the long radiative lifetime of ~1 μs, as illustrated below, the green emission can be ascribed to the radiative recombination STEs, which is prompted by the Jahn–Teller distortion of [SnBr_6_]^4−^ octahedra in 0D perovskite [31,32,33].

**Figure 3:**
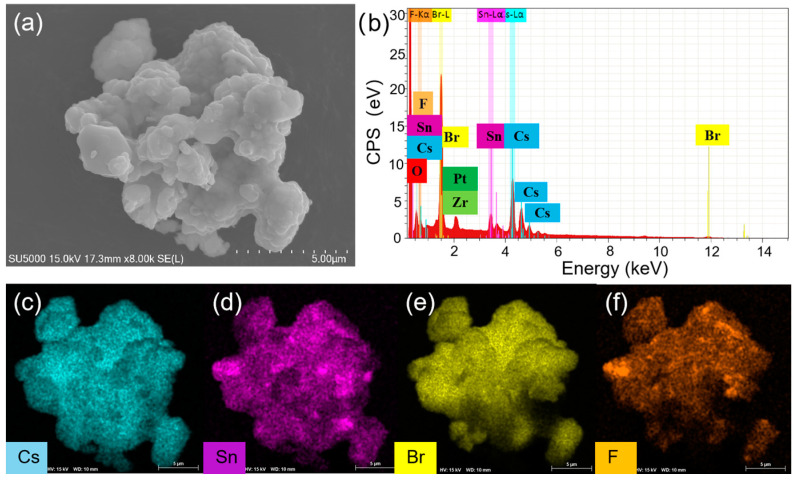
(**a**) SEM image, (**b**) EDS spectrum, and (**c**–**f**) EDS elemental maps of Cs, Sn, Br, and F for a typical pristine Cs_4_SnBr_6_ sample, respectively.

**Figure 4:**
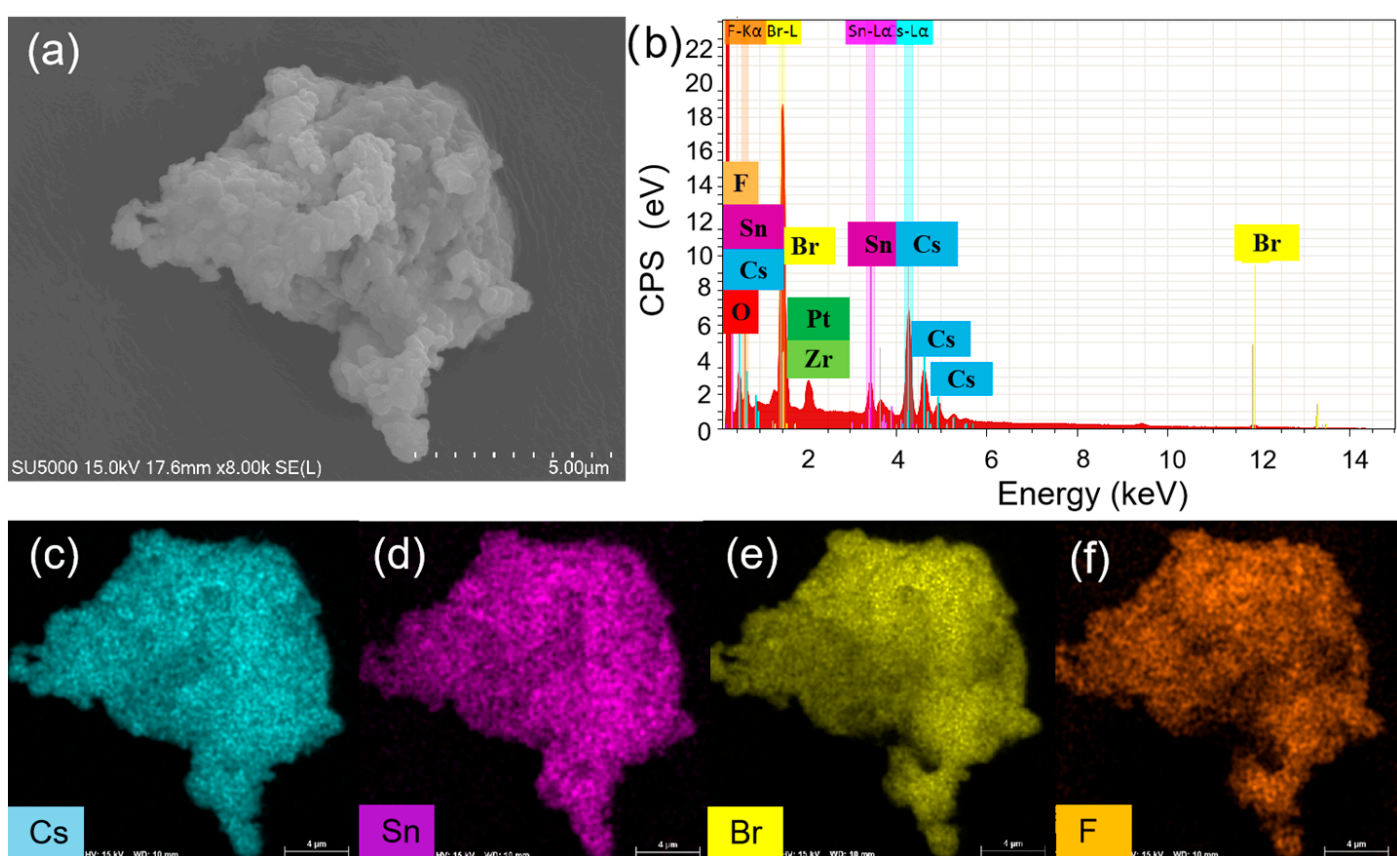
(**a**) SEM image, (**b**) EDS spectrum, and (**c**–**f**) EDS elemental maps of Cs, Sn, Br, and F for Cs_4_SnBr_6_ sample S-120 s annealed at an RTT temperature of 200 °C for 120 s, respectively.

To understand the enhanced PL, the structure and compositions of Cs_4_SnBr_6_ samples were evaluated via SEM and EDS, respectively. To characterize the sample morphology, a 20 nm-thick layer of Pt was coated onto the sample using magnetron sputtering. Figure 3a showcases the SEM image obtained from the pristine sample. The EDS spectrum reveals the presence of Cs, Sn, Br, and F elements in the SnF_2_-derived Cs_4_SnBr_6_, which are uniformly distributed, as demonstrated in the EDS mapping of Figure 3c–f. The Cs, Sn, Br, and F elements maintain this uniform distribution even after the sample was annealed at an RTT temperature of 200 °C for a duration of 120 s, as shown in Figure 4c–f. This result indicates that the elemental distribution in the sample remained unaltered when subjected to RTT at 200 °C for a duration of 120 s. From Figure 3b and Figure 4b, we also observed the presence of Zr and O elements, which originate from the zirconia balls utilized during ball milling.

**Figure 5:**
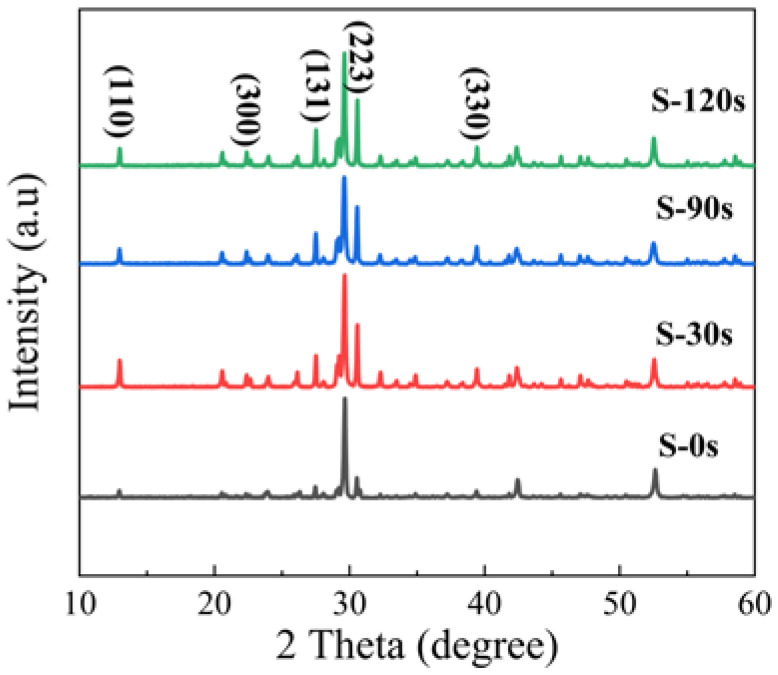
XRD patterns of the pristine Cs_4_SnBr_6_ sample and the samples annealed at an RTT temperature of 200 °C for 30 s, 90 s, and 120 s, respectively.

In Figure 5, we present the XRD patterns obtained for different samples. The XRD patterns of the pristine sample (S-0 s) and annealed samples reveal the coexistence of phases of Cs_4_SnBr_6_ and CsBr due to the incomplete consumption of CsBr powder precursors in the solid-state reaction. Apart from the prominent diffraction peak at 29.7°, attributed to the CsBr phase, we observe that the diffraction peaks of S-0 s from the Cs_4_SnBr_6_ phase are consistent with those reported for the SnF_2_-derived Cs_4_SnBr_6_ [19,34]. This consistency suggests that the substitution of Br^-^ with smaller F^−^ effectively suppresses the oxidation of Sn^2+^ in Cs_4_SnBr_6_. Notably, the diffraction peaks corresponding to crystal planes (110), (300), (131), (223), and (330) of the Cs_4_SnBr_6_ phase become more pronounced and well defined as the RTT time increases from 0 s to 120 s [34]. This strongly indicates an enhanced crystallinity of the Cs_4_SnBr_6_ powders after the RTT process.

**Figure 6:**
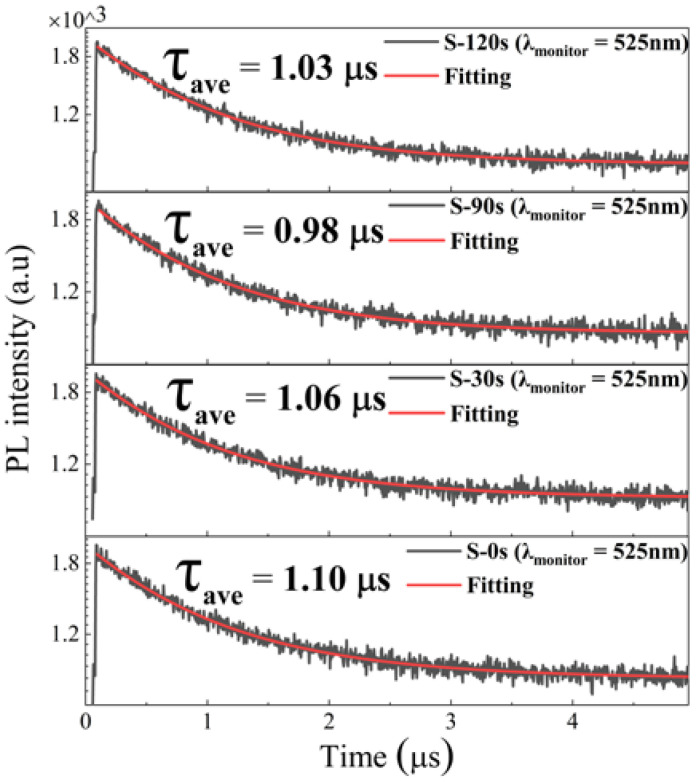
Time-resolved PL decay traces, captured at 525 nm, in both the pristine Cs_4_SnBr_6_ sample and the samples subjected to annealing at an RTT temperature of 200 °C for varying durations. Each measurement was conducted under an excitation wavelength of 375 nm, employing 70 ps excitation pulses from a laser.

To better comprehend the PL characteristics, we employed an excitation wavelength of 375 nm (facilitated by 70 ps excitation pulses from a laser) to measure the PL decay curves, as illustrated in Figure 6. The PL decay curve associated with the green emission yielded a sound fit when we employed a biexponential decay function [35]:

(1) $$I\left(t\right)=I_{0}+A_{1}exp\left(\frac{-t}{\tau_{1}}\right)+A_{2}exp\left(\frac{-t}{\tau_{2}}\right)$$

where *I*_0_ represents the background level, *τ*_1_ and *τ*_2_ represent the lifetimes of each exponential decay component, and *A*_1_ and *A*_2_ denote the corresponding amplitudes. The intensity-weighted averaged PL lifetimes are then determined by $\left(A_{1}*\tau_{1}^{2}+A_{2}*\tau_{2}^{2}\right)/\left(A_{1}*\tau_{1}+A_{2}*\tau_{2}\right)$ [35]. As depicted in Figure 6, the green emission in all samples exhibits a slow decay with a long radiative lifetime of approximately 1 μs. Notably, the lifetime remains nearly unchanged despite variations in the RTT conditions, even though the crystallinity of the Cs_4_SnBr_6_ samples improves after the RTT process. These findings suggest that the PL enhancement does not exclusively arise from a reduction in nonradiative recombination centers.

**Figure 7:**
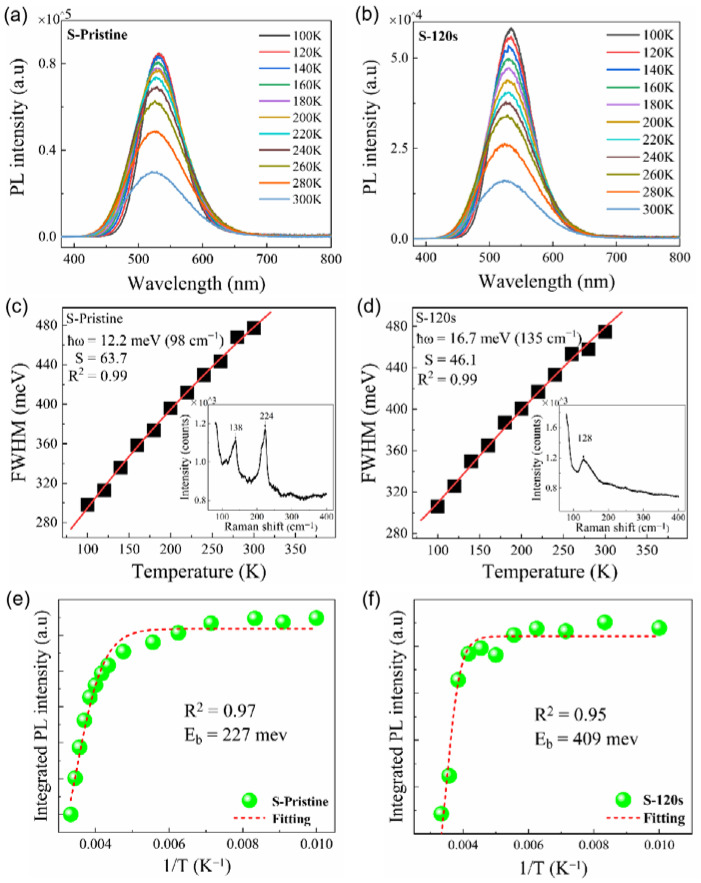
Temperature-dependent PL spectra measured in the range of 100 to 300 K for (**a**) pristine sample and (**b**) sample S-120 s annealed at an RTT temperature of 200 °C for 120 s. Temperature-dependent PL linewidth observed for (**c**) pristine sample and (**d**) sample S-120 s (solid symbols), and the fitting of the experimental data (red line) by using Equation (1). The insets in (**c**,**d**) show the Raman spectrum of pristine sample and sample S-120 s, respectively. Integrated PL intensities measured for (**e**) pristine sample and (**f**) sample S-120 s at different temperatures (green solid symbols). Also shown is the fitting of the corresponding experimental data (red dashed curve) of the pristine sample and sample S-120 s.

In order to gain insights into the influence of electron–phonon coupling, the temperature-dependent PL spectra of the samples were measured in the range of 100–300 K. As shown in Figure 7a,b, a decrease in temperature led to an increase in *PL* intensity and a reduction in the *FWHM* for both the pristine sample and sample S-120 s. According to the theory proposed by Stadler [36], the *FWHM* of the *PL* peak is closely related to the electron–phonon coupling and can be described by the following equation:

(2) $$FWHM\left(T\right)=2.36\sqrt{S}\hbar \omega \sqrt{\coth\frac{\hbar \omega }{2k_{B}T}}$$

where *S* is the Huang–Rhys factor, ħ*ω* is the energy of the phonon mode, *T* is the temperature, and k_B_ is Boltzmann’s constant. By fitting the temperature-dependent *FWHM* of the *PL* peaks using Equation (2), we can calculate the value of the Huang–Rhys factor S, which is commonly used to describe the exciton–phonon coupling [28]. For the pristine sample, it was found that the value of *S* is as large as 63.7 (see Figure 7c), which is significantly higher than that reported in Cs_4_SnBr_6_. Generally, a larger *S* value indicates a stronger electron–phonon coupling, which is more favorable for the formation of STEs. However, a high *S* value also implies an increasing probability of non-radiative recombination [24]. The Raman spectrum shown in the inset of Figure 7c reveals two dominant phonon modes, corresponding to the Sn-Br stretching vibrational modes at approximately 138 cm^−1^ and 224 cm^−1^ [37,38], respectively, which may be involved in the electron–phonon coupling and thus result in an *S* value as large as 63.7. After annealing the sample at an RTT temperature of 200 °C for 120 s, the value of S is significantly reduced to 46.1, which closely resembles that observed in Cs_4_SnBr_6_ [28]. From the inset of Figure 7c,d, one can see that the bimodal structure evolves into a single peak at 128 cm^−1^ upon annealing the sample at 200 °C for 120 s. This aligns well with the anticipated Sn-Br stretching vibrational mode, which is typically situated near 130 cm^−1^ in Cs_4_SnBr_6_ (see the inset of Figure 7d) [37,38]. This suggests that the electron–phonon coupling only involves the dominant phonon mode near 130 cm^−1^, which corresponds to the Sn-Br stretching vibrational mode near 130 cm^−1^. Given that thermal annealing is known to effectively diminish defects within the sample, we posit that the transition from a bimodal to a singular peak is a result of this defect reduction. This is in good agreement with the enhanced crystallinity of the Cs_4_SnBr_6_ powders after the RTT process, as evidenced by the X-ray diffraction (XRD) patterns (refer to Figure 5). Consequently, the observed deviation in the Raman mode energy from the expected ħ*ω* value of 12.2 meV (see the fitted data in Figure 7c) in the pristine sample may be attributable to inherent lattice defects. On another note, the *PL* QY of STEs highly depends on the exciton binding energy. The detrapping of STEs due to thermal activation results in a decreased radiative recombination rate. The exciton binding energy of STEs can be determined by analyzing the temperature-dependent integrated *PL* intensity (*I_PL_*) using the Arrhenius equation [33]:

(3) $$\;I_{PL\;}\left(T\right)=\frac{I_{PL\;}\left(T_{0}\right)}{1+\beta exp\left(-E_{b}/k_{B}T\right)}$$

where *I*_PL_(*T*_0_) is the integrated PL intensity at 100 K, *β* is a constant related to the density of radiative recombination centers, *k_B_* is Boltzmann’s constant, and *E_b_* is the exciton binding energy. Through fitting the experimental data with the Arrhenius equation, we can obtain the exciton binding energy *E_b_* of 227 meV and 409 meV for the pristine sample and sample S-120 s, respectively (see Figure 7e,f). It is noteworthy that the *E_b_* value in sample S-120 s substantially exceeds the 227 meV found in the pristine sample. This infers that the detrapping of STEs via thermal activation has been effectively suppressed in sample S-120 s, resulting in an augmented emission from the STEs. Therefore, based on the above analyses, we deduce that optimal electron–phonon coupling, compounded by the enhanced exciton binding energy elicited by the RTT, is responsible for the enhanced STE emission discerned in sample S-120 s.

## 4. Conclusions

In conclusion, a unique strategy employing rapid heat treatment to achieve efficient STE emission in Cs_4_SnBr_6_ zero-dimensional perovskite was demonstrated. We systematically investigated the impact of RTT on the PL properties of lead-free Cs_4_SnBr_6_. The results showed that RTT at 200 °C for 120 s significantly enhanced the emission from STEs, with the photoluminescence quantum yield increasing up to 64.7%. Through analysis of temperature-dependent PL spectra, Raman spectra, and PL decay traces, we propose that the improvement in photoluminescence is due to appropriate electron–phonon coupling and increased binding energies of STEs induced by the RTT. Our study offers a new approach for the development of efficient luminescent 0D tin-halide perovskites, which holds significant implications for future advances.

## Data Availability

Data underlying the results presented in this paper are not publicly available at this time but may be obtained from the authors upon reasonable request.
